# Aging changes in maxillary anterior teeth in untreated individuals: an observational longitudinal study

**DOI:** 10.1186/s40510-023-00478-z

**Published:** 2023-08-14

**Authors:** Gabriela Natsumeda, Felicia Miranda, Camila Massaro, José Roberto Pereira Lauris, Daniela Garib

**Affiliations:** 1 Department of Orthodontics, Bauru Dental School, University of São Paulo, Alameda Octávio Pinheiro Brisolla 9-75, Bauru, SP 17012-901 Brazil; 2 Department of Orthodontics, School of Dentistry, Federal University of Goiás, Goiânia, Goiás Brazil; 3 Department of Public Health, Bauru Dental School, University of São Paulo, Bauru, SP Brazil; 4 Department of Orthodontics, Hospital for Rehabilitation of Craniofacial Anomalies, University of São Paulo, Bauru, SP Brazil

**Keywords:** Normal occlusion, Adults, Maxillary anterior teeth, Aging

## Abstract

**Objective:**

The aging of the occlusion and tooth wears influence the smile design This study aimed at evaluating the aging changes of maxillary anterior teeth in nontreated subjects.

**Methods:**

The sample comprised dental models of 23 subjects (13 male, 10 female) with normal occlusions, taken at 13 (T1), 17 (T2) and 61 (T3) years of age. The following variables were measured in the maxillary anterior teeth using digital dental models: crown width/height proportion, anterior view width, crown angulation, gingival and incisal steps between central/lateral incisors and central incisors/canines. Interphase comparisons were evaluated using repeated measures analysis of variance followed by Tukey tests or Friedman tests. Sexual differences were evaluated using t tests (*P* < 0.05).

**Results:**

From 13 to 61 years of age, a decrease of crown width/height proportion (*P* = 0.008 and *P* =  < 0.001, for the lateral incisor and canines, respectively) and mesiodistal angulation (*P* =  < 0.001, *P* = 0.001 and *P* = 0.025 for the central incisor, lateral incisor and canines, respectively) of the maxillary anterior teeth were observed. The steps of the gingival margin and the incisal steps decreased with aging.

**Conclusions:**

From adolescence to late adulthood, untreated individuals with normal occlusions demonstrated changes in the maxillary anterior teeth that may impair the smile esthetics and attractiveness.

## Introduction

The seeking for orthodontic treatment by adult patients has been largely increased in the last decades [1]. An increased awareness of the need for adequate oral health and a greater expectation for dental esthetics from the society has occurred [1–3]. Approximately 49% of adults seeking orthodontic treatment have a chief complaint related to dental and facial esthetics [1]. Maturational changes specifically at the maxillary anterior teeth should be better understand for an adequate diagnosis and treatment plan for adult patients.

In general, clinical crown size changes with aging [4]. Subjects from 11 to 19 years of age showed an increase in the clinical crown length of anterior teeth [5]. A remarkable increase of crown height was observed from 13 to 60 years of age [6]. From adolescence to mature adulthood, the crown height increased 0.22, 0.76 and 1.50 mm for maxillary central incisors, lateral incisors and canines, respectively [6]. Mesiodistal crown width decreased as a result of interproximal attrition with aging [6, 7]. A 10-year follow-up study in Swedish women with initial age of 48 years showed a common tendency of crown lengthening due to significant extrusion of + 0.3 mm on average of the anterior maxillary teeth [8]. Gingival changes explain the clinical crown height increase with aging. From 6 to 16 years of age, gingival margin shows a continuous migration toward apical [9]. Adolescent patients at 15 years of age presented an apical migration of the gingival margin of 0.44 mm in 10 years of follow-up [10].

Despite the increase in crown height with aging, incisal tooth wear also occurs in adult patients [11]. During the aging process, erosive tooth wear caused by acid diet, attrition and abrasion occurs [12]. Occlusal/incisal surfaces displayed high wear scores in mature adults, especially in men [13]. The amount of tooth wear also influences the smile esthetics as the greater the tooth wear, the more unattractive is the smile [14]. A decrease in the maxillary incisors exposure for the upper lip is also expected with aging impairing smile esthetics [15]. A previous study showed that the maxillary incisor display for the upper lip decreased 3.6 mm from 17 to 61 years of age [16].

However, currently few evidence on the long-term gingival changes of the maxillary anterior teeth until the seventh decade of life is available. Positional changes on the maxillary anterior teeth are expected with the aging process. Understanding the aging changes of the anterior teeth is important to an adequate diagnosis and treatment planning of mature adult patients. In addition, maturational changes of the anterior teeth can have an influence on the long-term stability of orthodontic treatment. Therefore, the objective of this study was to investigate the positional and gingival changes of the maxillary anterior teeth expected from adolescence to late adulthood on digital dental models of untreated subjects.

## Material and methods

### Study design

This was an observational and longitudinal study. This study was approved by the Institutional Ethics Committee in Human Research at Bauru Dental School, University of São Paulo (process number #22082119.3.0000.5417).

### Participants and setting

**Fig. 1:**
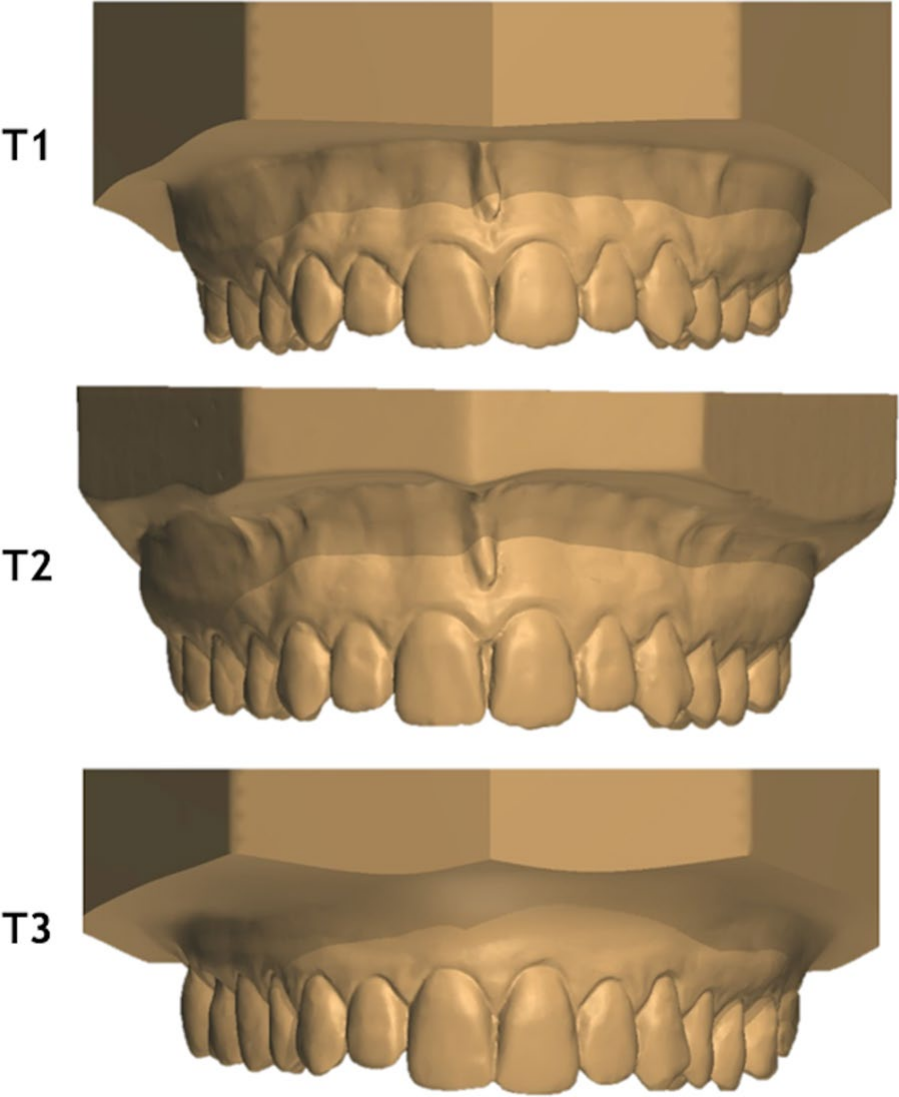
Maxillary digital dental models of a female subject from the sample at T1 (13 years), T2 (17 years) and T3 (61 years)

The sample comprised 23 White-Brazilian nontreated subjects (10 female, 13 male) with normal occlusion from the files of the Orthodontic Department at age 13 years (T1), 17 years (T2) and 61 years (T3), as shown in Fig. 1. The mean follow-up period was 47.98 years (SD, 0.95; range, 46.44–50.37). At T1, 82 individuals were selected according to the following inclusion criteria: normal occlusion [6, 16, 17] in the complete permanent teeth, dental Class I relationship, absence of crossbites, normal overjet and overbite, maximum of 2 mm of incisor crowding and no previous orthodontic treatment. The sample at T1 and T2 had been collected as a reference for facial growth studies in the Department of Orthodontics, Bauru Dental School, University of São Paulo. Patients were recalled at T3 for studying the aging of the normal occlusion. The exclusion criteria at T3 were history of orthodontic treatment and multiple or complete tooth losses. At T3, 23 patients out of 80 were found or agreed to participate.

### Variables and measurements

Dental models at the three-time points were digitized using an R700 3-dimensional (3D) scanner (3Shape, Copenhagen, Denmark). Dental model measurements were performed using OrthoAnalyzer three-dimensional software (3Shape) by a single examiner (G.N.). The investigator was initially trained for performing the analysis.

**Fig. 2:**
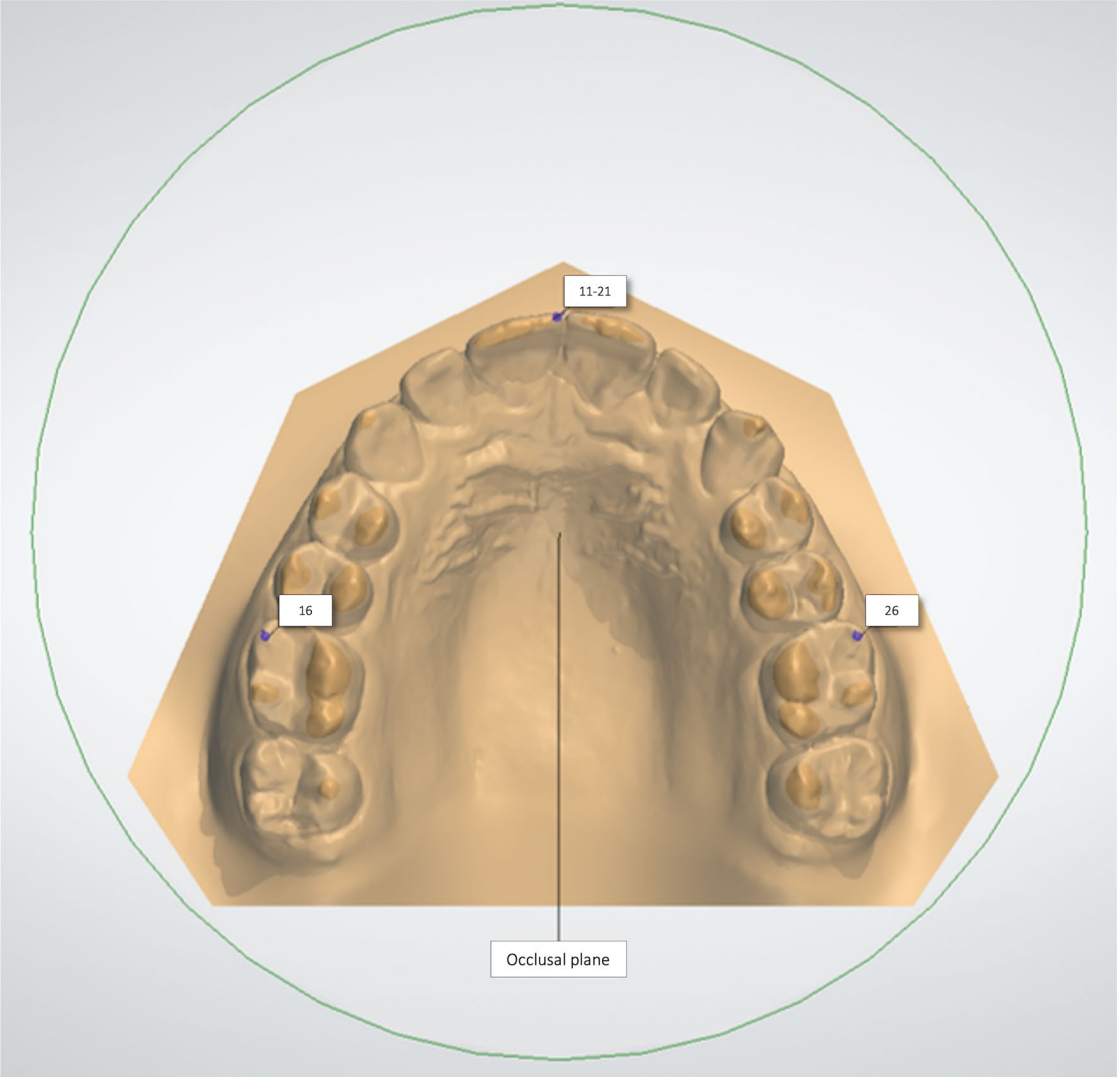
The occlusal plane was defined as a plane passing bilaterally through the tip of the first molar mesiobuccal cusp and through the mesioincisal point of the right central incisor

**Fig. 3:**
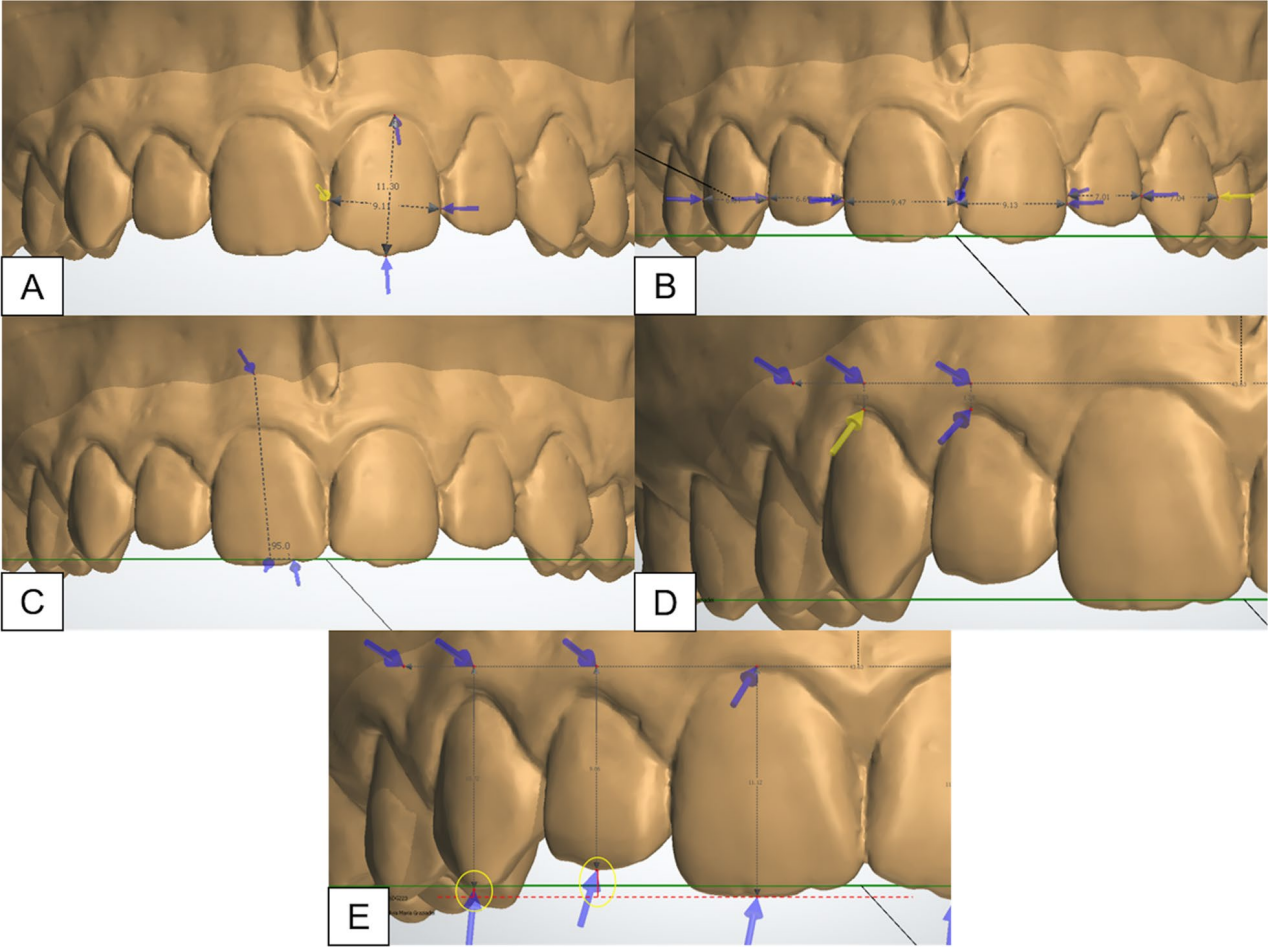
Measurements performed in the maxillary digital dental models: **A** Crown width/height proportion. The crown height measured from the gingival zenith to the incisal edge and crown width considered the maximum distance between the mesial and distal contact points; The width/height proportion was calculated after each value was recorded; **B** Mesiodistal dimension in the frontal view, with the occlusal plane parallel to the ground; **C** Crown angulation, measured using the occlusal plane and the long axis of the clinical crown; For **D** gingival and **E** incisal level between CI/LI and CI/C, a reference line was drawn parallelly to the occlusal plane and tangent to the most cervical portion of the gingival margin of the right central incisor was positioned. **D** Gingival level considered the distance between the reference line and the most cervical position of the gingival margin;** E** Incisal level was calculated using the distance between the reference line to the incisal edge of each tooth. Negative values indicated an apical position

The occlusal plane was used as reference for standardize the maxillary dental model position (Fig. 2). The following linear and angular measurements were performed in maxillary anterior teeth: (1) crown width/height proportion, (2) mesiodistal dimension in the frontal perspective (anterior view width), (3) crown mesiodistal angulation, gingival steps (4) and incisal steps (5) between the central and lateral incisors (CI/LI) and between the central incisors and canines (CI/C) (Fig. 3). The crown height was measured from the gingival zenith to the incisal edge [5]. The crown width dimension considered the maximum distance between the mesial and distal contact points of each tooth (Fig. 3A). The width/height proportion was calculated after each value was recorded.

The occlusal plane was positioned parallel to the ground for measuring the anterior view width, crown mesiodistal angulation, gingival and incisal steps (2–5). The mesiodistal dimension was measured in the frontal view (Fig. 3B). Crown mesiodistal angulation was evaluated using the occlusal plane and the long axis of the clinical crown. The actual angulation value was the obtained value minus 90º. (Fig. 3C). A reference line was drawn parallelly to the occlusal plane and tangent to the most cervical portion of the gingival margin of the right central incisor was positioned to evaluate the gingival and incisal steps. The gingival step considered the distance between the reference line and the most cervical region of the gingival margin of each tooth, allowing the calculation of CI/LI and CI/C gingival steps for each side (Fig. 3D). The distance between the reference line to the incisal edge of each tooth was measured to calculate the CI/LI and CI/C incisal step of each side (Fig. 3E). Negative values indicated an apical position.

### Study error

After one month from the first measurement, 30% of the sample were randomly selected and remeasured by the same examiner (G.N.). The intra-examiner reliability was assessed using intraclass correlation coefficients (ICC) [18] and the Bland–Altman method [19].

### Statistical analyses

Normal distribution of data was evaluated using Shapiro–Wilk test. The average right and left sides were used for statistics. Interphase comparisons were evaluated using ANOVA and Tukey tests or Friedman tests and Durbin-Conover tests. Comparisons between males and females were performed using t tests. All tests were performed with the Jamovi software (version 1.2.22), at *P* < 0.05. Data were analyzed blindly. A post hoc power analysis was also evaluated using the bilateral parametric test from the GPower software (Version 3.1.9.7, Heinrich-Heine-University, Dusseldorf, Germany).

## Results

Intraclass correlation coefficients varied from 0.86 to 0.98, indicating excellent intrarater agreement. The variable with the widest limit of agreement was the canine angulation ( − 3.61 and 4.66). The power of the sample was 99%, considering a mean change of 1.47 mm (SD = 1.38) in the canine crown height variable and a 5% significance level.

The aging process influenced most of the variables from T1 to T3 (Table 1). Crown width, mesiodistal angulation and gingival and incisal step decreased over time. On the other hand, clinical crown height increased. The anterior view width remained stable with no significant changes.

**Table 1:** Interphase comparison of anterior teeth measurements (ANOVA and Friedman tests)

Variable	Teeth	T1		T2		T3		*P*
		Mean	SD	Mean	SD	Mean	SD	
Width/Height Proportion (mm)	1	0.90	0.08	0.82	0.07	0.88	0.11	0.119^†^
	2	0.84^A^	0.08	0.78^B^	0.07	0.77^B^	0.13	0.008^₳^ *
	3	0.92^A^	0.10	0.81^B^	0.08	0.78^B^	0.12	< 0.001^₳^ *
Anterior view width (mm)	1	8.44	0.53	8.39	0.49	8.30	0.61	0.090^₳^
	2	6.34	0.48	6.29	0.34	6.22	0.46	0.738^†^
	3	6.50	0.60	6.47	0.55	6.40	0.57	0.433^₳^
Crown angulation (º)	1	4.95^A^	2.13	3.74^B^	1.80	2.01^C^	2.62	< 0.001^†^ *
	2	6.83^A^	3.00	5.13^B^	2.54	4.67^B^	4.05	0.001^₳^ *
	3	7.29^A^	5.02	5.19^B^	4.36	7.03^AB^	3.62	0.025^₳^ *
Gingival step (mm)	1–2	1.04^A^	0.50	0.91^A^	0.50	0.65^B^	0.53	0.002^₳^ *
	1–3	1.30^A^	0.83	0.83^B^	0.82	0.15^C^	0.90	< 0.001^₳^ *
Incisal step (mm)	1–2	− 0.67^A^	0.33	− 0.64^A^	0.33	− 0.40^B^	0.42	< 0.001^₳^ *
	1–3	0.21	0.69	0.42	0.51	0.44	0.60	0.138^₳^

Different letters in the same row indicate statistically significant differences by Tukey test or Durbin-Conover tests

1. central incisors; 2. lateral incisors; 3. canines. SD = Standard deviation

₳ repeated-measures ANOVA test; †. Friedman test

*Statistically significant at *P* < 0.05

From 13 to 17 years of age, lateral incisors and canines showed significant width/height proportion decrease (Table 1). Mesiodistal angulation decreased for all teeth. CI/C gingival step showed a significant decrease from T1 to T2.

From 17 to 61 years of age, crown mesiodistal angulation of central and lateral incisors continued to decrease (Table 1). Reductions in the CI/LI gingival and incisal step and in the CI/C gingival step were observed.

No sexual difference was observed, except for the canine angulation, which decreased more in women from T1 to T2 (Table 2).

**Table 2:** Male and female changes comparisons (t-tests)

Variable	Teeth	T2-T1				*P*	T3-T1				*P*	T3-T2				*P*
		Female		Male			Female		Male			Female		Male		
		Mean	SD	Mean	SD		Mean	SD	Mean	SD		Mean	SD	Mean	SD	
Width/Height Proportion (mm)	1	− 0.05	0.04	− 0.03	0.06	0.516	− 0.02	0.14	0.00	0.06	0.741	0.03	0.12	0.03	0.07	0.983
	2	− 0.05	0.04	− 0.05	0.06	0.775	− 0.08	0.12	− 0.05	0.12	0.621	− 0.02	0.11	0.00	0.13	0.715
	3	− 0.11	0.10	− 0.09	0.07	0.499	− 0.16	0.13	− 0.12	0.13	0.540	− 0.04	0.09	− 0.03	0.12	0.806
Anterior view width (mm)	1	− 0.03	0.21	− 0.06	0.14	0.635	0.02	0.39	− 0.27	0.28	0.066	0.05	0.46	− 0.20	0.23	0.091
	2	− 0.08	0.23	− 0.03	0.35	0.673	− 0.18	0.42	− 0.08	0.50	0.620	− 0.09	0.28	− 0.05	0.36	0.756
	3	0.03	0.18	− 0.06	0.33	0.433	− 0.01	0.26	− 0.16	0.51	0.421	− 0.04	0.35	− 0.10	0.49	0.772
Crown Angulation (º)	1	− 1.46	1.65	− 1.02	2.02	0.589	− 3.20	2.97	− 2.72	2.51	0.681	− 1.74	3.49	− 1.70	1.93	0.970
	2	− 1.95	2.41	− 1.50	2.06	0.639	− 3.50	2.85	− 1.12	3.12	0.074	− 1.55	2.48	0.38	3.05	0.117
	3	− 3.99	5.31	− 0.64	1.77	0.045*	− 1.81	4.11	0.93	3.67	0.106	2.17	3.72	1.57	3.50	0.697
Gingival step (mm)	1–2	− 0.11	0.34	− 0.14	0.43	0.861	− 0.36	0.57	− 0.41	0.68	0.832	− 0.24	0.44	− 0.27	0.54	0.898
	1–3	− 0.42	0.33	− 0.48	0.75	0.830	− 1.10	1.09	− 1.17	1.08	0.877	− 0.67	0.96	− 0.68	0.81	0.966
Incisal step (mm)	1–2	− 0.08	0.21	0.10	0.19	0.079	0.09	0.33	0.39	0.36	0.056	0.18	0.31	0.29	0.36	0.464
	1–3	0.08	0.37	0.29	0.60	0.334	− 0.03	0.65	0.42	0.63	0.104	− 0.11	0.62	0.13	0.51	0.317

*Statistically significant at *P* < 0.05

## Discussion

The maxillary anterior teeth are key factors for smile esthetics. For the best of our knowledge, this is the first study evaluating aging changes at the maxillary anterior region in nontreated subjects with normal occlusion. The changes promoted by aging in the smile esthetics were slight. However, when assessing anterior teeth esthetics, mild changes can cause a significant visual impact. Excellent intrarater agreement was found for all measurements. The examiner was trained for the measurements and only started the analysis when achieved a high precision. Long-term follow-ups of the occlusion in adults are challenging due to the difficulties in locating the subjects for follow-up appointments [6, 16, 17]. The follow-up time was approximately 50 decades in this study. The difficulties at T3 recruitment were changes in address, phone number and name due marriage. In addition, occlusal and dental status may change over time including tooth losses, prosthesis and dental restorations. In our study, the subjects who presented any of aforementioned changes in the maxillary anterior region were excluded. Previous studies analyzing this same sample of untreated patients demonstrated the maturational occlusal and cephalometric changes [6, 16, 17]. The main changes observed after 47 years of follow-up were mandibular crowding, decrease in the overbite, changes in the maxillary second molar position, increase in the clinical crown length, discoloration, increases in the maxillary and mandibular anterior displacement, and increases in the facial and ramus height [6, 16, 17].

From adolescence to late adulthood, the width/height proportion decreased in lateral incisors and canines from T1 to T2. The explanation is that clinical crown height increase and the mesiodistal crown width decrease during aging [6]. This result is in agreement with previous studies that reported interproximal wear and reductions of mesiodistal tooth size [6, 20, 21]. We speculate that no change in width/height proportion occurred in the central incisors due to a greater amount of incisal wear that compensates the changes in the gingival level. The anterior view width showed slight but no significant decrease from T1 to T3. This slight reduction can be explained by the expected mesiodistal tooth size decrease that occurs with the aging process [6].

Crown mesiodistal angulation significantly decreased for all anterior teeth with aging. Central incisors showed a progressive angulation decrease from adolescence until the seventh decade of life. On the other hand, the lateral incisors and canines showed a decrease of the mesiodistal angulation only from 13 to 17 years of age. A previous study using digital dental models of subjects with a mean age of 70 years found a mesiodistal angulation of 1.26º for maxillary central incisors, 5.46º for lateral incisors and 7.84º for canines [22]. The uprighting of maxillary anterior teeth during aging might collaborate with the absence of late incisor crowding in the maxillary arch observed in nontreated individuals [6].

The gingival step between central and lateral incisors decreased by 0.4 mm from T1 to T3. The gingival step between the central incisors and canines also decreased by 1.2 mm during the observational time. At T3, the gingival margin of the central incisor and canines were almost at the same level. These changes are probably related to an apical displacement of the gingival margin of these teeth [10]. The apical migration of the gingival margin of lateral incisors and canines should have been greater compared to the central incisors explaining the decrease in the gingival steps. A previous study performed in subjects from 22 to 84 years of age showed that gingival recessions in the maxilla were more common at canines and lateral incisors [23]. Thinner buccal bone thickness and less distance between cementoenamel junction and bone crest are expected for anterior teeth with gingival recessions [23]. If gingival recessions are more frequent in maxillary canines and lateral incisors compared to central incisors, the natural apical migration of the gingival margin during aging is also expected to be greater in these regions.

**Fig. 4:**
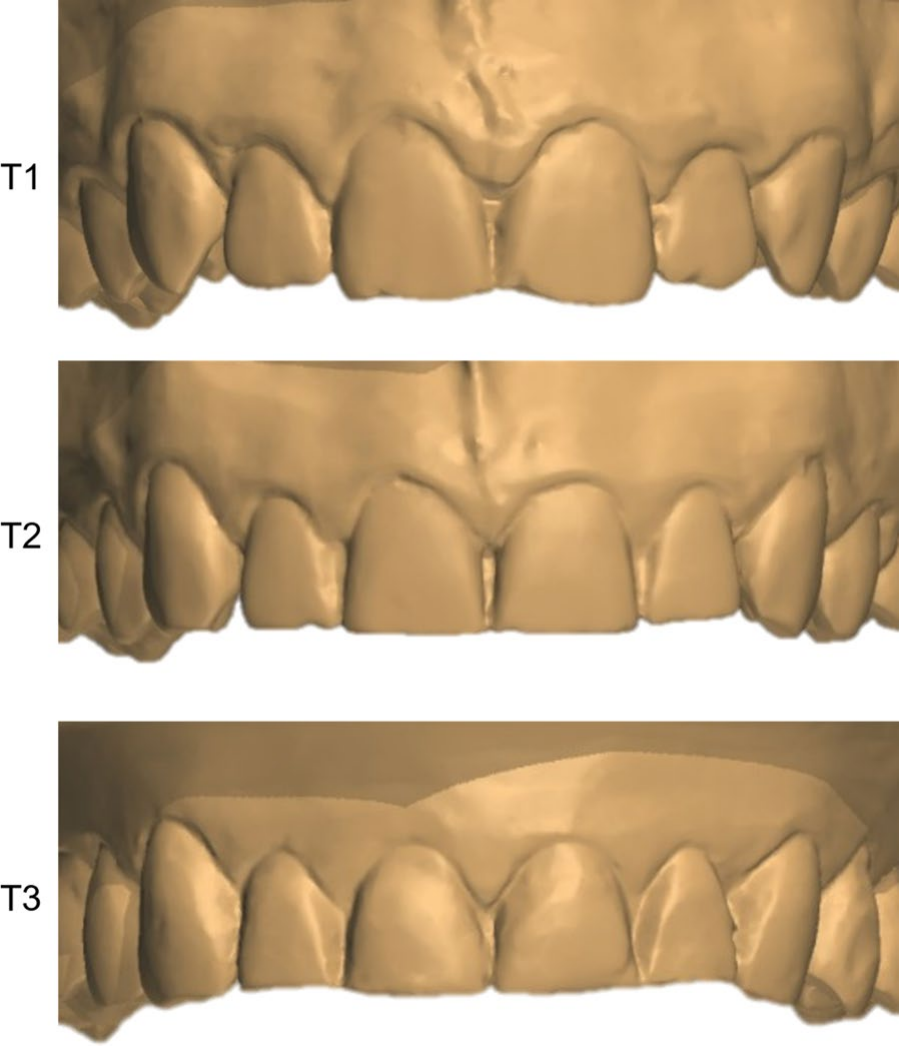
Male subject at 13 (T1), 17 (T2) and 61 years of age (T3). Observe the reductions of the incisal step between the central and lateral incisors

The incisal step between central and lateral incisors observed during adolescence was lost until late adulthood (Fig. 4). These findings were expected due to incisal tooth wear that occurs during aging [11, 24]. Anterior teeth show a significant level of tooth wear caused by the anterior guidance [25]. Throughout life, teeth are exposed to physical injuries as parafunctional habits and regular mastication and chemical exposure including acidic drinks/foods and gastric reflux, which contributes to tooth wear [26, 27]. The overbite reduction and the edge-to-edge incisor relationship are common features observed during aging, especially in men [6, 17]. Consequently, the incisal edge of the maxillary incisors becomes at the same level and may cause a smile impairment. Previous studies on the influence of vertical position of central incisors on the smile esthetics showed that no step between the maxillary incisors was considered unattractive [28, 29]. The incisal edge of the maxillary anterior teeth affected by tooth wear can be augmented in the adulthood aiming a smile esthetic improvement [30]. In addition, the incisal tooth wear and loss of the incisal step between central and lateral incisors might have an influence on the anterior functional guidance increasing the surface of contact between maxillary and mandibular incisors. However, the anterior functional guidance was not assessed in any of the timepoints of this study.

From adolescence to early adulthood, the maxillary canines demonstrated a greater mesiodistal upright movement in females than males (Table 2). These differences might be explained by the late facial growth pattern. In women, the mandible rotates down and backward during aging while in men the mandible displaced forward and downward [16, 31]. The greater upright movement of maxillary canine upright in women might represent a sagittal compensation for a more convex profile. Small dental adjustments may occur to maintain an adequate occlusion in front of skeletal changes during the aging process.

### Limitations

The limitation of this study is the lack of information on diet and parafunctional habits, factors that could have influenced anterior teeth wear. Another limitation of this study is the small sample size. The number of patients included in the study can be justified by the longitudinal design of this study, which offers considerable challenges in data collection. However, this study provided important information about aging covering maturation over 48 years, following a same sample longitudinally. Future studies should compare the aging changes in maxillary anterior teeth of untreated subjects and orthodontically treated patients.

## Conclusions

From 13 to 61 years of age, the following changes occurred in the maxillary anterior teeth:

- The crown width/height proportion and mesiodistal teeth angulation decreased;
- The gingival step between central and lateral incisors and between central incisors and canines decreased;
- The incisal step between the central and lateral incisors decreased.

## Data Availability

The datasets used and/or analysed during the current study are available from the corresponding author on reasonable request.

## Abbreviations

CI/LI: Central and lateral incisors
CI/C: Central incisors and canines
SD: Standard deviation
